# GPX4 and vitamin E cooperatively protect hematopoietic stem and progenitor cells from lipid peroxidation and ferroptosis

**DOI:** 10.1038/s41419-021-04008-9

**Published:** 2021-07-15

**Authors:** Qian Hu, Yifan Zhang, Huiling Lou, Zexian Ou, Jin Liu, Wentao Duan, Hao Wang, Yuanlong Ge, Junxia Min, Fudi Wang, Zhenyu Ju

**Affiliations:** 1 Key Laboratory of Regenerative Medicine of Ministry of Education, Institute of Aging and Regenerative Medicine, Jinan University, Guangzhou, China; 2 Department of Geriatrics, National Key Clinical Specialty, Guangzhou First People’s Hospital, School of Medicine, South China University of Technology, Guangzhou, China; 3 Department of Nutrition, Precision Nutrition Innovation Center, School of Public Health, Zhengzhou University, Zhengzhou, China; 4 The First Affiliated Hospital, School of Public Health, Institute of Translational Medicine, Zhejiang University School of Medicine, Hangzhou, China

**Keywords:** Cell biology, Physiology, Stem-cell research

## Abstract

Ferroptosis, a newly defined mode of regulated cell death caused by unbalanced lipid redox metabolism, is implicated in various tissue injuries and tumorigenesis. However, the role of ferroptosis in stem cells has not yet been investigated. Glutathione peroxidase 4 (GPX4) is a critical suppressor of lipid peroxidation and ferroptosis. Here, we study the function of GPX4 and ferroptosis in hematopoietic stem and progenitor cells (HSPCs) in mice with *Gpx4* deficiency in the hematopoietic system. We find that *Gpx4* deletion solely in the hematopoietic system has no significant effect on the number and function of HSPCs in mice. Notably, hematopoietic stem cells (HSCs) and hematopoietic progenitor cells lacking *Gpx4* accumulated lipid peroxidation and underwent ferroptosis in vitro. α-Tocopherol, the main component of vitamin E, was shown to rescue the *Gpx*4-deficient HSPCs from ferroptosis in vitro. When *Gpx4* knockout mice were fed a vitamin E-depleted diet, a reduced number of HSPCs and impaired function of HSCs were found. Furthermore, increased levels of lipid peroxidation and cell death indicated that HSPCs undergo ferroptosis. Collectively, we demonstrate that GPX4 and vitamin E cooperatively maintain lipid redox balance and prevent ferroptosis in HSPCs.

## Introduction

Continuous replenishment of the hematopoietic system depends on hematopoietic stem cell (HSC) self-renewal and differentiation into progenitors and eventually mature blood cells [1]. Most HSCs remain quiescent under homeostatic conditions but are stimulated to proliferate and differentiate upon encountering intrinsic or extrinsic stresses [2]. Reactive oxygen species (ROS), mainly generated endogenously during mitochondrial oxidative phosphorylation, are involved in the determination of HSC fate. Low levels of ROS are essential to maintain the quiescent status of HSCs, while high levels of ROS abolish quiescence, limit lifespan, and induce cell death [2, 3].

Scavenger networks have been developed to protect cells from excess ROS. Among them, glutathione peroxidase 4 (GPX4), a kind of selenoenzyme, specifically reduces lipid peroxidation by utilizing glutathione (GSH). Indeed, as the major hydroperoxide scavenger, GPX4 is a vital suppressor of ferroptosis [4, 5], a newly defined type of programmed cell death caused by iron-dependent lipid peroxidation accumulation [6, 7]. Failure of the lipid peroxide reducing system caused by GSH depletion or *Gpx4* deficiency results in ferroptosis [8], which has been implicated in a plethora of diseases involving multiple tissues and cells. Mice with loss of *Gpx4* did not survive during embryonic development [9]. Conditional induction of *Gpx4* knockout in adult mice impaired the integrity of various organs, such as the brain and kidney [4, 10]. Cell type-specific *Gpx4* depletion led to cell death and dysfunction in the retina, brain, liver, and reproductive tissue [11–15]. Moreover, GPX4 was revealed to be essential in hematopoietic cells including T cells, erythroid precursors, and myeloid cells [16–18]. However, the function of GPX4 and the influence of ferroptosis on hematopoietic stem and progenitor cells (HSPCs) remain unclear.

Vitamin E is a well-known lipophilic antioxidant that naturally exists in foods such as plant seeds, vegetables, and eggs. α-Tocopherol (α-Toc), the major component of vitamin E, reduces cell lipid peroxides and prevents ferroptosis [4, 12]. In contrast, hydrophilic antioxidants such as NAC are ineffective against ferroptosis induced by *Gpx4* deficiency. Moreover, dietary supplementation with vitamin E alleviates phenotypes resulting from *Gpx4* deficiency in endothelial cells, hepatocytes, T cells, myeloid cells, and reticulocytes [15, 16, 18–20].

Here we found that *Gpx4* depletion in the hematopoietic system alone had no significant effect on the number and function of HSPCs in mice. Intriguingly, HSPCs lacking *Gpx4* accumulated lipid peroxidation and dramatically underwent ferroptosis in vitro. Further investigation indicated that Vitamin E/α-Toc is the crucial factor leading to the discrepant fate of HSPCs. *Gpx4* knockout mice fed a vitamin E-depleted diet showed a reduced number of HSPCs and impaired function of the hematopoietic system. High levels of total ROS and lipid peroxidation indicated that HSPCs underwent ferroptosis. In summary, our results showed that GPX4 cooperates with vitamin E, maintaining the balance of lipid redox and preventing ferroptosis in HSPCs.

## Results

### GPX4 deficiency results in HSPCs ferroptosis in vitro

**Fig. 1:**
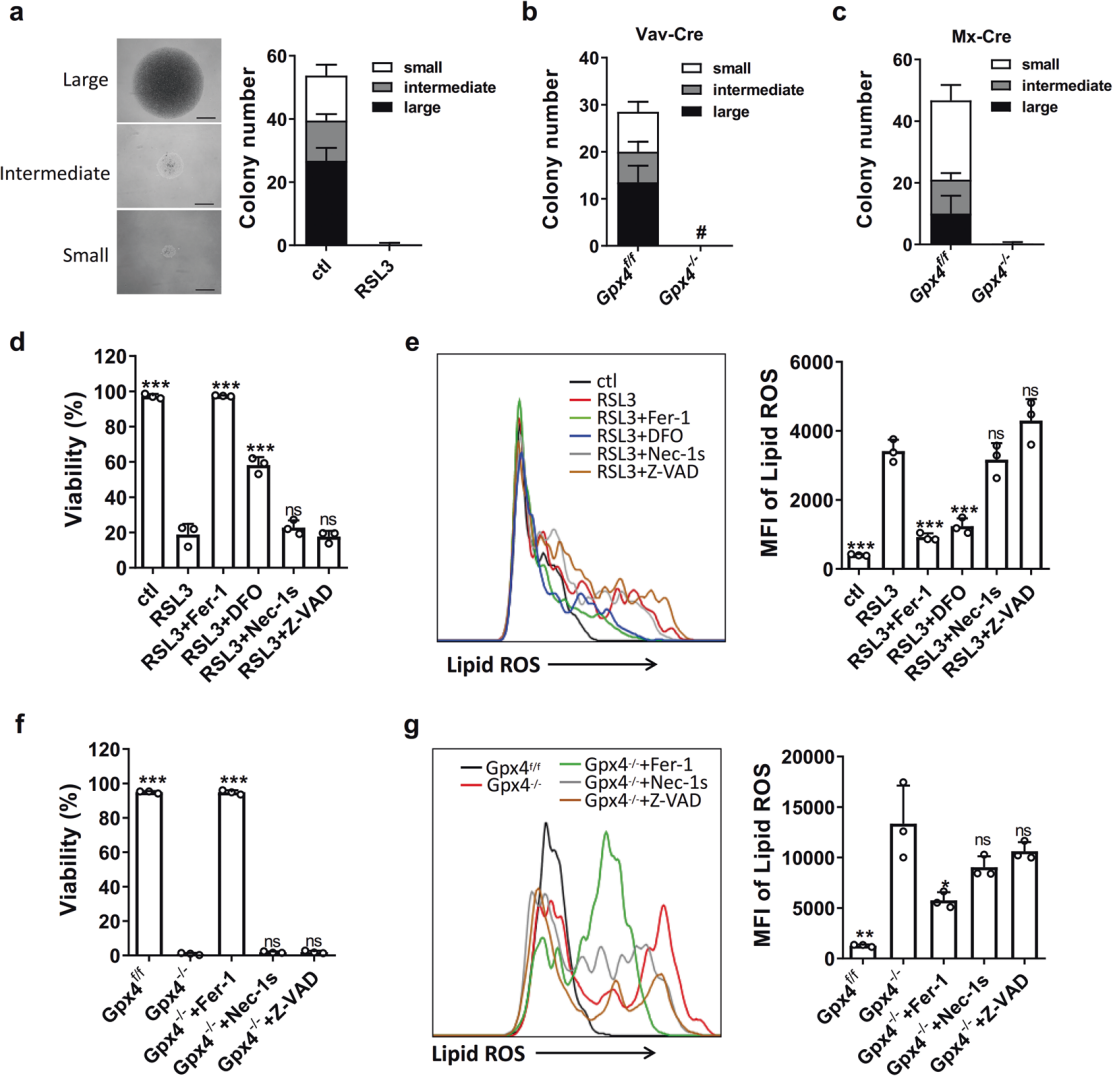
GPX4 deficiency induces HSPC ferroptosis in vitro. **a** LT-HSC single cells were sorted and cultured in a medium with or without RSL3 for 14 days. Colony numbers were counted (*n* = 4 mice). Scale bars = 0.5 mm. LT-HSCs derived from the *Gpx4*^flox/flox^ mice and *Gpx4*^flox/flox^ Vav-Cre mice (**b**) or the pIpC-treated *Gpx4*^flox/flox^ Mx-Cre mice (**c**) were tested with single-cell colony-forming assay as in (**a**) (*n* = 2 Vav-Cre mice or 4 Mx-Cre mice). **d** The viability of LSK cells isolated from the wild-type mice after 48 h of culture with the indicated drugs (*n* = 3 mice). **e** LSK cells isolated from the wild-type mice were cultured with the indicated drugs for 24 h, and lipid ROS were measured with C11-BODIPY and detected by flow cytometry (*n* = 3 mice). **f** The viability of LSK cells isolated from the *Gpx4*^flox/flox^ mice or the *Gpx4*^flox/flox^ Vav-Cre mice after 48 h of culture with the indicated drugs (*n* = 3 mice). **g** LSK cells isolated from the *Gpx4*^flox/flox^ mice or the *Gpx4*^flox/flox^ Vav-Cre mice were cultured with the indicated drugs for 24 h, and lipid ROS were measured with C11-BODIPY and detected by flow cytometry (*n* = 3 mice). Data are the mean ± SD. (ns not significant, *P < 0.05, **P < 0.01, ***P < 0.001 vs. the RSL3 group in (**d**, **e**), vs. the *Gpx4*^*−/−*^ group in (**f, g**).

HSCs rapidly proliferate and differentiate to generate single-cell colonies when they are cultured in vitro. For determination of whether GPX4 is essential for HSC function, individual long-term HSCs (LT-HSCs) from wild-type mice were sorted and treated with RSL3, a widely used GPX4 inhibitor. Strikingly, RSL3 completely blocked the colony formation of LT-HSCs (Fig. 1a). To exclude the off-target effects of RSL3, we generated *Gpx4*^flox/flox^ Vav-Cre mice in which *Gpx4* was specifically knocked out in the hematopoietic system and *Gpx4*^flox/flox^ Mx-Cre mice in which *Gpx4* was depleted in the hematopoietic system by pIpC treatment [21]. Then, the LT-HSCs isolated from the *Gpx4*^flox/flox^ Vav-Cre mice and the pIpC-treated *Gpx4*^flox/flox^ Mx-Cre mice were evaluated by a single-cell colony-forming assay. Similar to the RSL3-treated LT-HSCs, the *Gpx4*-depleted (*Gpx4*^*−/−*^ LT-HSCs lost the ability to produce single-cell colonies (Fig. 1b, c). Next, we assessed the susceptibility of different types of HSPCs to RSL3. We observed that LT-HSCs and short-term HSCs (ST-HSCs) were more resistant to RSL3 than multipotent progenitors (MPPs) and granulocyte-macrophage progenitors (GMPs) (Supplementary Fig. 1a). Consistently, the expression levels of GPX4 in the LT-HSCs and ST-HSCs were higher than those in the MPPs and GMPs (Supplementary Fig. 1b). These results suggest that GPX4 is indispensable for HSC function in vitro.

Given that GPX4 is a vital suppressor of ferroptosis, we wondered whether HSPCs undergo ferroptosis in the absence of GPX4. Hence, lineage^−^sca1^+^c-kit^+^ (LSK) cells isolated from the wild-type (Fig. 1d, e) or *Gpx4*-depleted mice (Fig. 1f, g) were cultured in vitro and treated with different drugs. Both the RSL3-treated LSK cells and the *Gpx4*-depleted LSK cells accumulated lipid peroxides and underwent cell death within two days (Fig. 1d−g). Moreover, neither Nec-1s nor Z-VAD affected cell viability, excluding the involvement of necroptosis and apoptosis. In contrast, the lipophilic antioxidant ferrostatin 1 (Fer-1) and the iron chelator deferiprone (DFO) reduced lipid peroxide levels and suppressed cell death (Fig. 1d−g). We noticed that although lipid peroxidation was diminished, nearly half of the RSL3-treated LSK cells failed to be rescued by DFO (Fig. 1d). Considering the essential biological function of iron in cells, we speculated that DFO may be toxic to HSPCs as they are highly dependent on iron. Indeed, after treatment with DFO alone, both LSK cells and GMPs died in vitro (Supplementary Fig. 1c, d). These results demonstrate that GPX4 deficiency leads to LSK cell ferroptosis in vitro. Furthermore, a similar scenario was observed in GMPs: both RSL3-treated and *Gpx4*-depleted GMPs accumulated lipid peroxides and succumbed to ferroptosis (Supplementary Fig. 1e–h). In summary, the above results suggest that HSPCs undergo ferroptosis in vitro when GPX4 is inhibited or depleted.

### *Gpx4* deletion does not affect homeostasis of the hematopoietic system

**Fig. 2:**
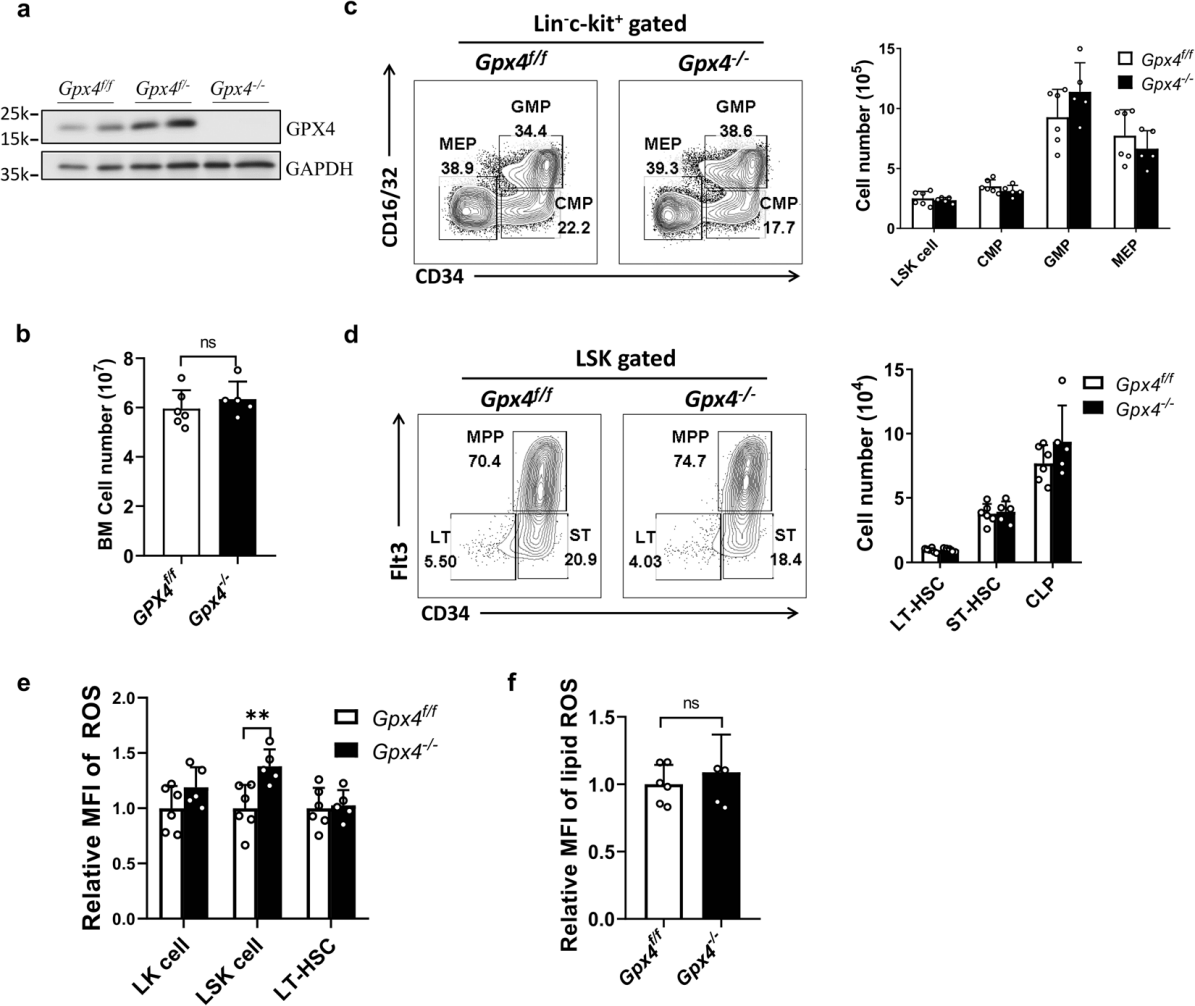
Gpx4 deletion does not affect hematopoietic system homeostasis in the *Gpx4*^flox/flox^ Vav-Cre mice. **a** *Gpx4* deletion in the blood cells of the *Gpx4*^flox/flox^ Vav-Cre was verified by Western blots. **b** The BM cell numbers were counted. **c**, **d** The numbers of HSPCs were measured by flow cytometry. Left panels: representative flow cytometric plots. Right panels: statistical data. **e** The relative MFI of ROS in LK cells, LSK cells, and LT-HSCs (CD34^−^Flt3^−^LSK cells). **f** The relative MFI of lipid peroxidation in BM cells was detected by C11-BODIPY. *N* = at least five mice in each group. Data are the mean ± SD. (ns not significant, **P < 0.01).

It has been reported that GPX4 is indispensable in embryonic development and the integrity of multiple tissues [9–11, 13]. To further explore the function of GPX4 in HSPCs in vivo, we studied the phenotype of HSPCs in mice in which *Gpx4* was knocked out in the hematopoietic system. *Gpx4* depletion in the hematopoietic system of the *Gpx4*^flox/flox^ Vav-Cre mice was confirmed by Western blots (Fig. 2a). Surprisingly, no significant difference was observed between the *Gpx4*^flox/flox^ Vav-Cre mice (*Gpx4*^*−/−*^) and the *Gpx4*^flox/flox^ mice (*Gpx4*^*f/f*^) in the numbers of total bone marrow (BM) cells (Fig. 2b) and HSPCs (Fig. 2c, d) including LT-HSCs, ST-HSCs, MPPs, LSK cells, GMPs, and common lymphoid progenitors (CLPs). Moreover, *Gpx4* ablation had no significant impact on either the ROS levels of LT-HSCs and lineage^−^c-kit^+^ (LK) cells or the lipid ROS levels of BM cells (Fig. 2e, f). Thus, the HSPC phenotype in the *Gpx4*^flox/flox^ Vav-Cre mice at homeostasis was not affected by *Gpx4* depletion. Given that *Gpx4* deletion is detrimental in numerous tissues and Gpx4 was deleted at the embryonic stage in the *Gpx4*^flox/flox^ Vav-Cre mice, it is possible that some compensatory mechanism conferred HSPC resistance to the *Gpx4* deficiency. To rule out this possibility, we constructed *Gpx4*^flox/flox^ Mx-Cre mice, in which *Gpx4* deletion in the hematopoietic system was induced by pIpC treatment (Supplementary Fig. 2a). Similar to that in the *Gpx4*^flox/flox^ Vav-Cre mice, the HSPC phenotype was not impaired in the *Gpx4*^flox/flox^ Mx-Cre mice treated with pIpC (Supplementary Fig. 2b–g). These results demonstrate that *Gpx4* deficiency does not affect homeostasis of the hematopoietic system in mice.

### *Gpx4* deficiency does not affect HSC function in vivo

**Fig. 3:**
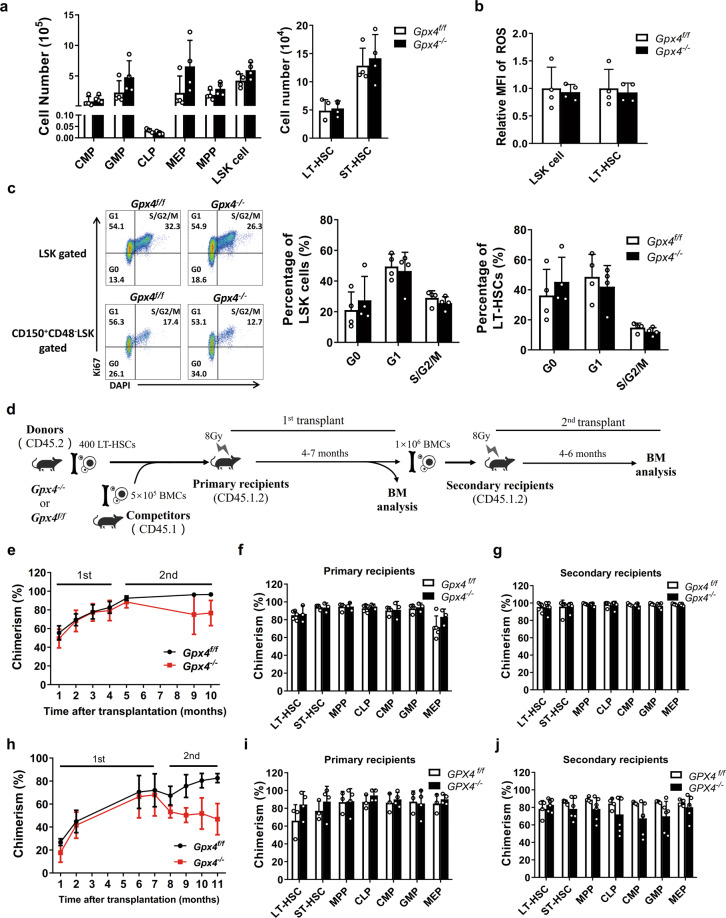
Gpx4 deficiency does not affect HSC function in vivo. **a** The number of HSPCs, **b** the relative MFI of ROS in LSK cells and LT-HSCs, and **c** the cell cycle of LSK cells and LT-HSCs from the pIpC-treated *Gpx4*^flox/flox^ Mx-Cre mice 7 days after 5-FU treatment were measured. **b** The experimental schematic for serial competitive transplantation with LT-HSCs. **e** The chimerism of peripheral blood cells, **f** the chimerism of HSPCs at the 4th month after primary transplantation, **g** the chimerism of HSPCs at the 6th months after secondary transplantation in the recipient mice receiving LT-HSCs from the *Gpx4*^flox/flox^ Vav-Cre mice. **h** The chimerism of peripheral blood cells, **i** the chimerism of HSPCs at the 7th month after primary transplantation, **j** the chimerism of HSPCs at the 4th month after secondary transplantation receiving LT-HSCs from the *Gpx4*^flox/flox^ Mx-Cre mice. *N* = at least three recipient mice in each group in (**e**−**j**). Data are the mean ± SD.

During homeostasis, most HSCs remain quiescent, with low metabolic activity and low ROS levels [2]. We wondered whether GPX4 is essential for HSCs under stress in vivo. The *Gpx4*-deleted mice were treated with 5-fluorouracil (5-FU) to remove cycling HSCs, which are susceptible to 5-FU. The remaining quiescent HSCs then proliferated and differentiated to restore the hematopoietic system. Nine days post 5-FU treatment, we found no significant difference in the number of HSPCs in the *Gpx4*-deleted mice compared with the wild-type mice (Fig. 3a). Neither the levels of ROS nor the proliferation rates of LSK cells and LT-HSCs were affected by *Gpx4* depletion (Fig. 3b, c). Similar results were found in mice 22 days post 5-FU treatment (Supplementary Fig. 3a, b). These results suggest that the hematopoietic system recovered normally in the *Gpx4*-deleted mice.

To further investigate the function of HSCs in vivo, we conducted competitive HSC transplantation to evaluate the long-term rebuilding capacity of HSCs in the *Gpx4*-depleted mice. LT-HSCs obtained from the *Gpx4*^*f/f*^ mice or *Gpx4*^*−/−*^ mice (CD45.2) together with BM cells obtained from competitor mice (CD45.1) were transplanted into recipient mice (CD45.1.2), whose hematopoietic system was destroyed by a lethal dose of X-ray irradiation (Fig. 3d). Once transplanted, donor-derived HSCs home to bone marrow and replenish the hematopoietic system in recipient mice. However, no obvious difference in the chimerism rates of peripheral blood cells within four months (Fig. 3e) was observed even though the T cell chimerism rates were lower in the mice that received *Gpx4*-depleted HSCs (Supplementary Fig. 3c). Furthermore, the chimerism rates of various HSPCs especially CLPs in the primary recipient at the fourth month were not affected by *Gpx4* depletion (Fig. 3f), indicating that the difference in T cell chimerism rates was not due to the difference in HSC differentiation. Indeed, it has been reported that *Gpx4*-deficient T cells have intrinsic defects in homeostatic balance maintenance and cell expansion upon stress [16]. After secondary transplantation, there was still no significant difference in the chimerism rates of the HSPCs between the recipients of the *Gpx4*-deficient and *Gpx4*-sufficient LT-HSCs (Fig. 3g). Likewise, transplantation with LT-HSCs derived from the pIpC-treated *Gpx4*^flox/flox^ Mx-Cre mice resulted in no significant change in the chimerism rates of the *Gpx4*-deficient HSPCs during either primary or secondary transplantation (Fig. 3h−j). Thus, our results show that *Gpx4* deficiency does not affect HSC self-renewal and differentiation in vivo, which is quite distinct from the scenario in vitro.

### α-Toc rescues HSPCs from ferroptosis ex vivo

What leads to the entirely different destiny of the *Gpx4*-deficient HSPCs in vivo and ex vivo? The levels of oxygen are much higher in the culture medium than in the bone marrow niche, which may inflict more ROS stress on HSPCs [22]. We then tested whether ferroptosis is mediated by higher oxygen levels in HSPCs in vitro. The results revealed that RSL3-induced ferroptosis in both LSK cells and GMPs, was only partially relieved when they were cultured at low oxygen levels. In addition, the oxygen levels did not affect the viability of either the *Gpx4*-deleted LSK cells or the *Gpx4*-deleted GMPs (Supplementary Fig. 4a, b). Consistently, the lipid ROS levels of HSPCs were not reduced under low levels of oxygen (Supplementary Fig. 4c, d). These results suggest that oxygen levels are not the main reason for ferroptosis in the *Gpx4*-deficient HSPCs.

**Fig. 4:**
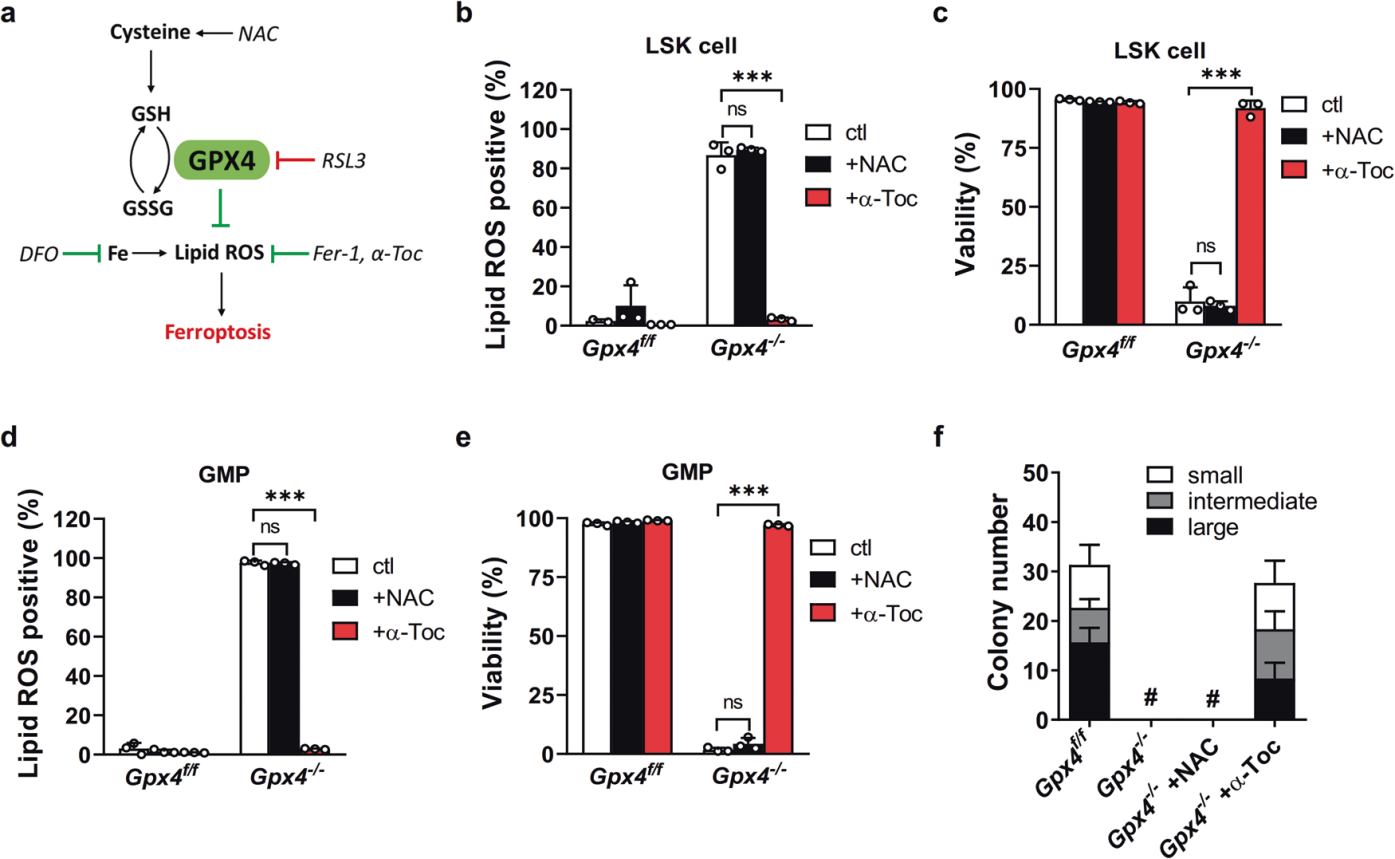
α-Toc rescues HSPCs from ferroptosis ex vivo. **a** Schematic illustration of the ferroptosis pathway and the targets of different drugs. Ferroptosis was triggered by iron-dependent accumulation of lipid peroxidation. GPX4 makes use of GSH to reduce lipid peroxidation and inhibit ferroptosis. Ferroptosis can be induced by the GPX4 inhibitor RSL3 and inhibited by the iron chelator DFO or lipophilic antioxidants such as Fer-1 and α-Toc. N-Acetyl-L-cysteine (NAC) is the precursor of cysteine, which promotes GSH synthesis. LSK cells (**b**, **c**) or GMPs (**d, e**) from the *Gpx4*^*f*lox/flox^ Vav-Cre mice were cultured with NAC or α-Toc. The viability and lipid ROS levels of these cells were measured (*n* = 3 mice). **f** LT-HSCs derived from the *Gpx4*^flox/flox^ Vav-Cre mice were tested with single-cell colony-forming assay in a medium containing NAC or α-Toc (*n* = 3 mice). # indicates no colony was formed. Data are the mean ± SD. (ns not significant, ***P < 0.001).

It has been reported that dietary vitamin E alleviates phenotypes resulting from lipid peroxidation in T cells and reticulocytes [16, 17]. α-Toc, the prominent component of vitamin E, is a lipophilic antioxidant and has been proven to reduce lipid peroxidation and block ferroptosis in vitro [4, 12] (Fig. 4a). We suspected that α-Toc can inhibit HSPC ferroptosis in vitro. Certainly, we observed that α-Toc, instead of N-acetyl-L-cysteine (NAC), a hydrophilic antioxidant, significantly reduced lipid ROS and inhibited ferroptosis in both LSK cells and GMPs (Fig. 4b−e). Furthermore, α-Toc endowed *Gpx4*-deficient LT-HSCs with the ability to generate colonies (Fig. 4f). Thus, α-Toc protects HSPCs from ferroptosis ex vivo.

### Deficiency of both GPX4 and vitamin E induces HSPC ferroptosis and impairs homeostasis of the hematopoietic system in vivo

**Fig. 5:**
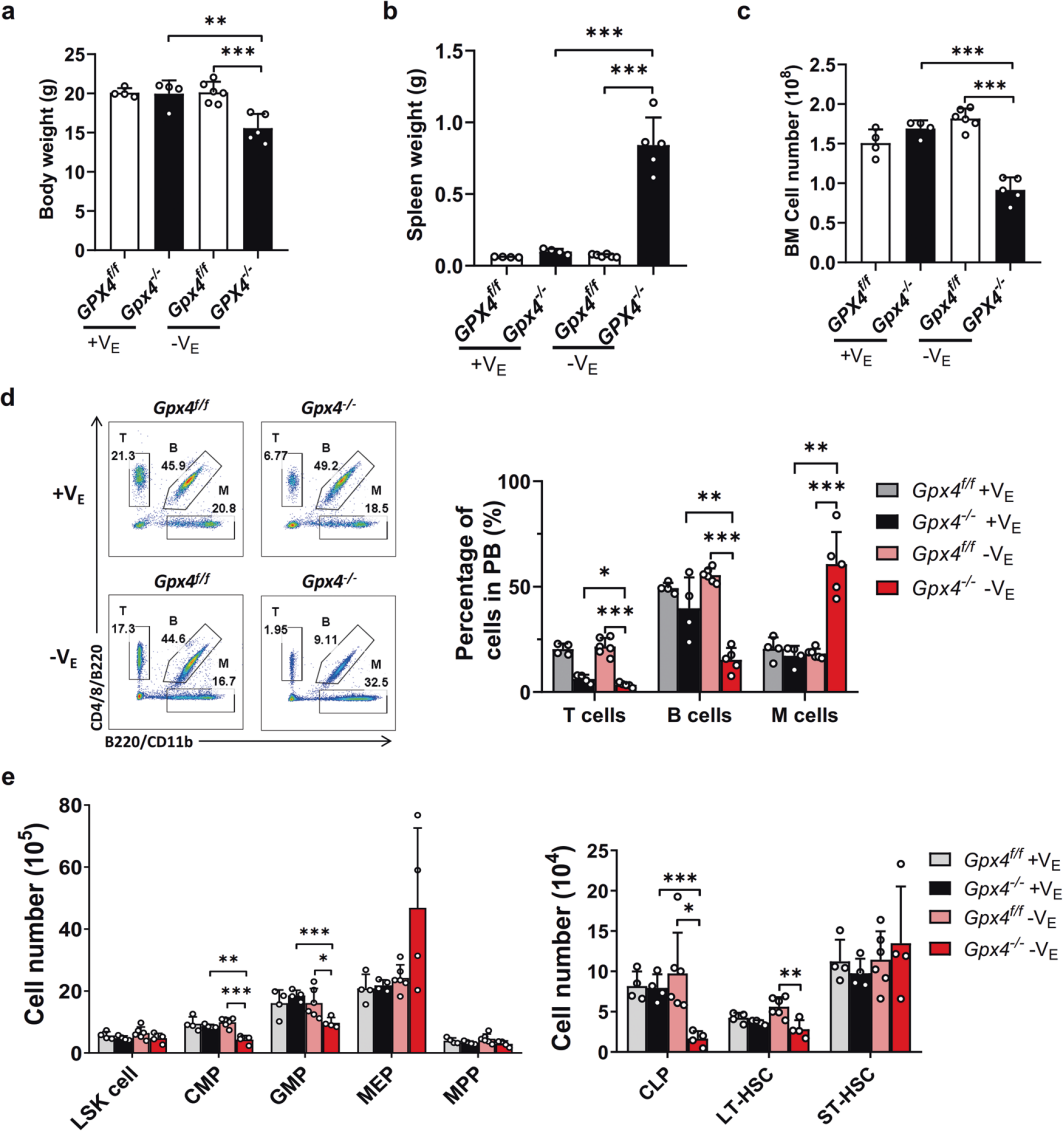
Deficiency of both GPX4 and vitamin E impairs hematopoietic system homeostasis. **a** The body weight, **b** spleen weight, and **c** total BM cell counts of the *Gpx4*^flox/flox^ Vav-Cre mice were measured after 3 weeks of a V_E_-depleted diet. **d** Left panel: representative flow cytometric plots of the T cell, B cell, and myeloid cell groups in peripheral blood. Right panel: statistical data. **e** The number of HSPCs in the *Gpx4*^flox/flox^ Vav-Cre mice after 3 weeks of a V_E_-depleted diet. *N* = at least four mice in each group. Data are the mean ± SD. (*P < 0.05, **P < 0.01, ***P < 0.001).

Vitamin E is absent in the cell culturing medium but is abundantly supplied from experimental animal food. Given that α-Toc rescues HSPCs in vitro, we hypothesized that dietary vitamin E is critical to protect the *Gpx4*-deficient HSPCs from ferroptosis in vivo. In previous experiments, mice were fed natural ingredient diets containing ≥120 IU/kg vitamin E. To verify our hypothesis, we then fed mice a fixed formulation diet (containing 75 IU/kg vitamin E) or a vitamin E-depleted diet for 3 weeks. Strikingly, we found that combined depletion of *Gpx4* and vitamin E led to the loss of body weight, splenomegaly, and reduced BM cell number (Fig. 5a−c). Moreover, in peripheral blood, the proportion of both T cells and B cells decreased significantly while the proportion of M cells increased (Fig. 5d). More importantly, the numbers of LT-HSCs, CMPs, GMPs, and CLPs decreased significantly upon the removal of *Gpx4* and vitamin E together (Fig. 5e). However, *Gpx4* deletion alone did not impair the HSPC phenotype, consistent with previous results (Fig. 5a−e). Similarly, the depletion of vitamin E alone did not affect the HSPC phenotype (Fig. 5a−e). These data suggest that vitamin E and GPX4 cooperate to maintain hematopoietic system homeostasis.

**Fig. 6:**
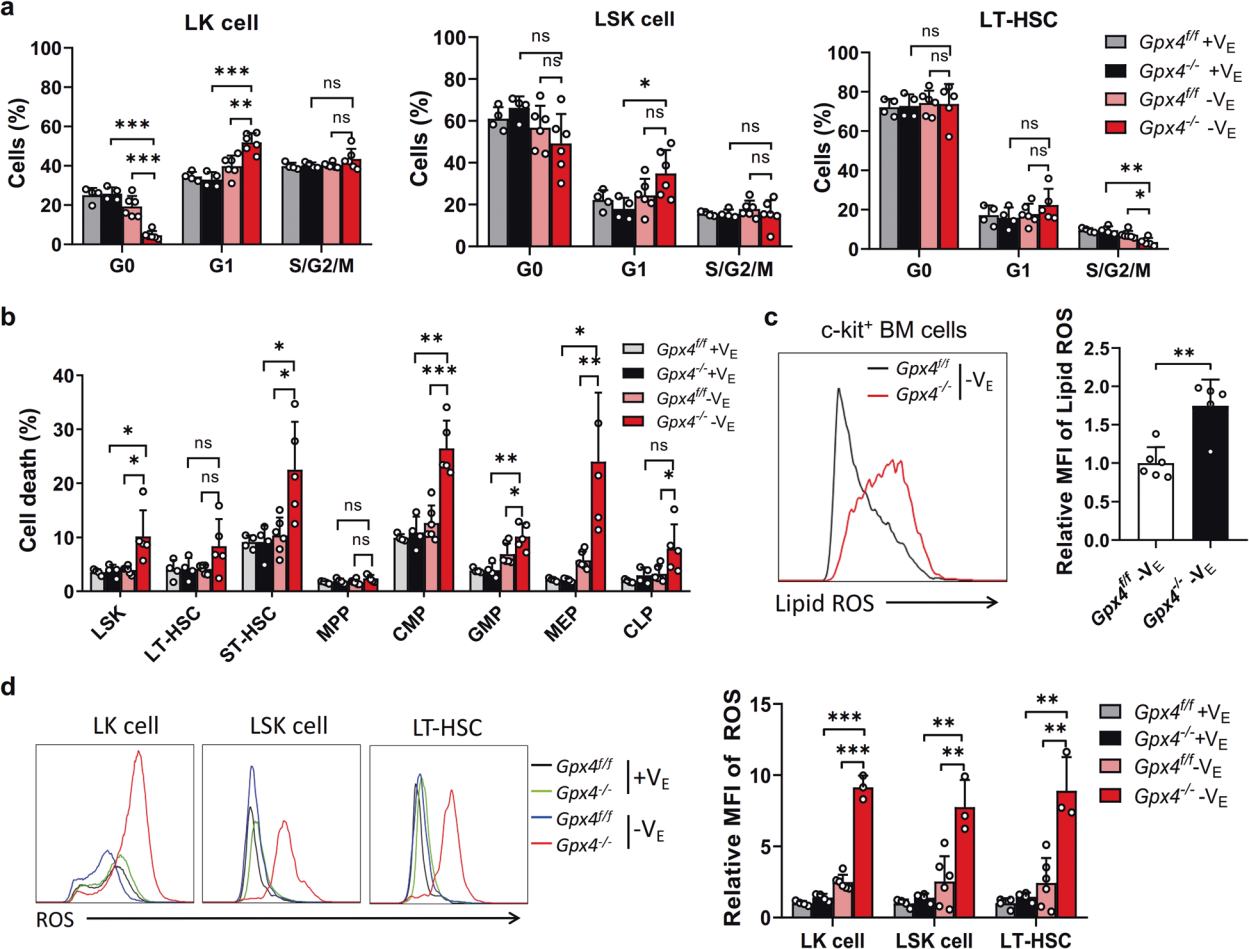
Deficiency of both GPX4 and vitamin E results in HSC ferroptosis in vivo. **a** The cell cycle of LK cells, LSK cells, and LT-HSCs in the *Gpx4*^flox/flox^ Vav-Cre mice after 3 weeks of a V_E_-depleted diet. **b** The HSPCs in the *Gpx4*^flox/flox^ Vav-Cre mice after 3 weeks of a V_E_-depleted diet were determined to be DAPI negative. **c** Left panel: representative flow cytometric plots of lipid ROS in c-kit^+^ cells of the *Gpx4*^flox/flox^ Vav-Cre mice after 3 weeks of a V_E_-depleted diet. Right panel: statistical data of the relative MFI of lipid ROS. **d** Left panel: representative flow cytometric plots of ROS in LK cells, LSK cells, and LT-HSCs of the *Gpx4*^flox/flox^ Vav-Cre mice after 3 weeks of a V_E_-depleted diet. Right panel: statistical data of the relative MFI of ROS. *N* = at least three mice in each group. Data are the mean ± SD. (ns not significant, *P < 0.05, **P < 0.01, ***P < 0.001).

To determine, how combined depletion of Gpx4 and vitamin E impaired hematopoietic system homeostasis, we first detected the cell cycle of HSPCs. In the absence of *Gpx4* and vitamin E, no significant change in the percentage of LT-HSCs and LSK cells in G0 phase was found (Fig. 6a), indicating that the proliferation of these cells was not blocked. In contrast, significant death of HSPCs was found in the mice deficient in both *Gpx4* and vitamin E (Fig. 6b), which was responsible for the decreased number of these HSPCs. Next, we observed enhanced lipid ROS levels in c-kit^+^ cells (Fig. 6c). Moreover, the ROS levels dramatically increased in LK cells, LSK cells, and LT-HSCs of the *Gpx4*-deficient mice fed vitamin E depleted chow (Fig. 6d). Taken together, our results reveal that GPX4 and vitamin E cooperatively protect HSPCs from ferroptosis in vivo.

## Discussion

Exponential progress has been made in the field of ferroptosis in recent years in identifying ferroptosis regulators, unveiling their pathological function, and developing drugs or therapies [23]. The relevance of ferroptosis to diseases related to multiple tissues has been well documented [24]. However, previous reports have mainly focused on differentiated cells, leaving the role of ferroptosis in stem cells poorly understood. Here, we focused on HSPCs, which are the most widely studied adult stem cells. We showed that GPX4 deficiency in HSPCs caused by inhibitors or genetic deletion leads to lipid peroxidation and ferroptosis in vitro. In contrast, dietary vitamin E is sufficient to protect HSPCs from ferroptosis and prevent hematopoietic system impairment in the *Gpx4*-depleted mice. Our work identified ferroptosis in HSPCs for the first time and demonstrates the cooperative effects of *Gpx4* and vitamin E on the maintenance of HSPC function and the homeostasis of the hematopoietic system.

As a critical lipid peroxidation scavenger in cells, GPX4 is a key regulator of ferroptosis and is essential to the functional integrity of various tissues [25]. Previous studies reported that *Gpx4* is involved in erythropoiesis and that *Gpx4* deficiency in the hematopoietic system results in RIP3-dependent necroptosis of erythroid precursors [17]. Another report showed that myeloid-specific deficiency of *Gpx4* drove pyroptosis during polymicrobial sepsis [18]. In contrast, our work mainly focuses on HSPCs, the top of the hematopoietic pyramid, and we found that *Gpx4*-deficient HSPCs tend to undergo ferroptosis. These diverse outcomes of *Gpx4* deficiency in different kinds of hematopoietic cells indicate diverse regulation of lipid peroxidation and cell death types.

Long-term ex vivo expansion of HSCs has long been a critical challenge for HSC transplantation, which is a curative therapy for numerous blood disorders [26, 27]. Here we found GPX4 is essential for HSC survival ex vivo, implicating the importance of lipid peroxidation scavenging. Therefore, whether lipophilic antioxidants such as a-Toc may improve the efficiency of HSC expansion ex vivo is worth future study.

Vitamin E is well known as a hydrophobic antioxidant and an essential nutrient. Vitamin E deficiency is associated with neuron abnormalities, ataxia, and hemolytic anemia [28]. Dietary supplementation with vitamin E ameliorates membrane fragility of red blood cells and thus prevents hemolysis [29]. More importantly, it has been previously reported that vitamin E can compensate for GPX4 deficiency in some tissues including endothelial cells, hepatocytes, T cells, myeloid cells, and reticulocytes [15, 16, 18–20]. Similarly, our results highlight the importance of dietary vitamin E in protecting HSPCs from lipid peroxidation and ferroptosis in addition to the GPX4/GSH system. Given that vitamin E is an effective ferroptosis inhibitor supplied from normal animal chow, caution needs to be taken to evaluate the influence of vitamin E on the phenotypes related to lipid peroxidation and ferroptosis.

The potential physiological application of ferroptosis has been highlighted in recent years. Ferroptosis inhibitors including vitamin E, have the potential to alleviate diseases resulting from ferroptosis such as ischemic perfusion injury, neurodegeneration, and acute renal injury. Recently, ferroptosis was reported to be involved in radiation-induced cell death [30–32]. This finding may partially explain the radioprotective function of vitamin E and selenium in the hematopoietic system [33, 34]. Nevertheless, it has also been reported that vitamin E may promote tumor metastasis [35] and increase the risk of cancer [36]. Indeed, ferroptosis is considered cancer-suppressive. Many types of cancer especially therapy-resistant cancer cells are sensitive to GPX4 deficiency[37, 38]. The development of cancer therapies based on the induction of ferroptosis alone or combined with other therapeutic approaches has shown promise [39]. Thus, on one side, there may be a potential dosing window to kill malignant hematopoietic cells by targeting GPX4, leaving healthy HSPCs intact. On the other side, it needs to be elucidated whether tumor cells or malignant hematopoietic cells can hijack vitamin E against ferroptosis-inducing therapy.

## Materials and methods

### Mice

C57BL/6 WT mice were purchased from Beijing HFK BioScience Company (Beijing, China). *Gpx4*^flox/flox^ mice (CD45.2) were a kind gift from Professor Fudi Wang. *Gpx4*^flox/flox^ mice were crossed with Vav-Cre mice and Mx-Cre mice to generate the *Gpx4*^flox/flox^ Vav-Cre mice and *Gpx4*^flox/flox^ Mx-Cre mice, respectively. For *Gpx4* deletion, *Gpx4*^flox/flox^ Mx-Cre mice were intraperitoneally injected with 20 mg/kg pIpC (Sigma) every other day for two weeks. CD45.1/45.2 mice and CD45.1 mice on a C57BL/6 background were used as competitor and recipient mice, respectively, in the competitive transplantation assay. Mice were fed natural ingredient diets containing ≥120 IU/kg vitamin E. A fixed formulation diet with or without 75 IU/kg vitamin E (Beijing HFK BioScience Company, Beijing, China) was fed to the mice involved in the vitamin E depletion experiments. For 5-FU treatment, mice were intraperitoneally injected with 150 mg/kg 5-FU (Sigma). The hematopoietic phenotype was analyzed 9 and 22 d after treatment. Two to four-month age mice of both sexes were utilized, and all experimental protocols were approved by the Animal Care and Ethics Committee of Jinan University.

### Flow cytometry and cell sorting

For flow cytometric analysis, cells were stained and labeled with fluorophore-conjugated antibodies purchased from BD Bioscience, Invitrogen, and Biolegend. All the antibody clones and fluorescent labels for FACS and flow cytometry are listed in Supplementary Table 1. After staining, red blood cells were eliminated by lysis buffer (BD Biosciences) and analyzed on a BD Fortessa system. For FACS, c-kit^+^ cells were separated from BM cells with anti-APC microbeads (Miltenyi) and then stained with antibodies for surface markers. HSPCs were then sorted by BD Arial III. The data were analyzed using FlowJo software.

### Cell culture

For in vitro experiments, HSPCs were cultured in DMEM/F12 medium (Gibco) supplemented with 10% fetal bovine serum (Gibco), 20% BIT9500 (StemCell), 2 mM L-glutamine (Gibco), penicillin/streptomycin, 5 × 10^–5^ M β-ME, 10 ng/ml stem cell factor (PeproTech), 10 ng/ml thrombopoietin (PeproTech), and 15 ng/ml interleukin-3 (PeproTech). For the single-cell colony-forming assay, single LT-HSCs were sorted in 96-well plates and cultured for 14 days in the medium described above. The numbers and sizes of colonies were assessed under microscopy.

### HSC transplantation

For the HSC primary transplantation assay, 400 LT-HSCs from donor mice were injected via the orbital route into irradiated (8 Gy X-ray) recipient mice together with 5 × 10^5^ BM cells from competitor mice. For the second transplantation, 1 × 10^6^ BM cells from primary recipient mice were transplanted into irradiated (8 Gy X-ray) secondary recipient mice.

### Measurement of ROS

After surface marker staining, BM cells were stained with 20 μM DCFDA following the manual of the DCFDA/H2DCFDA-Cellular ROS Assay Kit (Abcam) at 37 °C in the dark for 30 min. Cells were then washed and analyzed by flow cytometry.

### Measurement of lipid ROS

The cells to be tested were incubated with 5 μM C11-BODIPY (Life Technology) at 37 °C for 30 min, washed, and detected by flow cytometry. The levels of lipid ROS were measured in the FITC channel.

### Analysis of cell cycle

For cell cycle analysis, cells were fixed by incubating with 120 μl of BD Cytofix/Cytoperm buffer at RT for 30 min in the dark. The cells were then washed with BD Perm/Wash Buffer and permeabilized with 0.4% Triton for 2–3 min. After washing, the cells were stained with Ki67 monoclonal antibody for 40 min at RT in the dark. Finally, the cells were stained with 1 mg/ml DAPI before flow cytometry.

### Q-PCR

Total RNA was purified from HSPCs using the RNAqueous^®^-Micro Kit (Life Technology) and then reverse-transcribed using PrimeScript™ RT Master Mix (TaKaRa). Real-time PCR was performed on a QuantStudio 6 Flex system (Applied Biosystems). The amount of target mRNA was normalized to that of β-actin mRNA. The gene expression quantities were determined according to the relative *C*_*t*_.

### Western blot

For Western blot, BM cells were lysed in RIPA buffer. Then, the proteins were run on standard 12% SDS-PAGE gels, transferred onto PVDF membranes (Bio-Rad), and blocked with TBST containing 5% milk for 1 h at RT. The membranes were then incubated with primary antibodies overnight at 4 °C, washed with TBST, incubated with secondary antibodies for 1 h at RT, and detected on an Amersham Imager 600 System (GE). Primary antibodies against GPX4 (Abcam, ab125066) and GAPDH (CST, 5174) and secondary antibodies against rabbit IgG (CST, 7074) and mouse IgG (CST, 7076) were used. The data were analyzed using ImageJ.

### Statistics

The sample sizes were described in the figure legend and were determined as at least three biologically independent animals according to previous studies performed by our group. No exclusions of data were made that would significantly impact the results or conclusions. Animals with the same genotype and gender and similar age were randomly assigned to experimental groups. Investigators were not blinded during the group allocation during the experiment. Analysis of flow cytometric data was performed using FlowJo version 10. GraphPad Prism 8 was used for statistical analysis. The results are shown as the mean ± SD. For data that were fitted with a Gaussian distribution, unpaired Student’s two-tailed *t*-test was used to determine the statistical significance. For data that were not fitted with a Gaussian distribution, the Mann−Whitney test was used. (ns not significant, * *P* < 0.05, **P < 0.01, ***P < 0.001.)

## Supplementary information

Supplementary figure 1

Supplementary figure 2

Supplementary figure 3

Supplementary figure 4

Supplementary Table 1
