# Supplementary material for: GPX4 and vitamin E cooperatively protect hematopoietic stem and progenitor cells from lipid peroxidation and ferroptosis
Source: Cell Death Dis. 2021 Jul 15;12(7):706. doi: 10.1038/s41419-021-04008-9 (PMC8282880; doi:10.1038/s41419-021-04008-9)
Supplement: Supplementary file 5 — Supplementary Table 1 [file 41419_2021_4008_MOESM5_ESM.docx]

**Supplementary table 1. Antibody clones and fluorescent labels for FACS sorting and analysis：**

| **Fluorescence** | **Antibody** | **Clone** | **Company** |
| --- | --- | --- | --- |
| FITC | CD4 | RM4-5 | Biolegend |
| FITC | CD8 | 53-6.7 | Biolegend |
| FITC | B220 | RA3-6B2 | Biolegend |
| FITC | CD34 | RAM34 | BD Biosciences |
| FITC | Ki67 | 11F6 | Biolegend |
| FITC | Brdu | 3D4 | Biolegend |
| Pecp-cy5.5 | CD48 | HM48-1 | Invitrogen |
| Pecp-cy5.5 | CD45.1 | A20 | Invitrogen |
| Pecp-cy5.5 | IL-7R | A7R34 | Invitrogen |
| PE | 45.2 | 104 | Biolegend |
| PE | Flt3 | A2F10 | Invitrogen |
| PE-Cy7 | Sca1 | E13-161.7 | Biolegend |
| APC | c-kit | 2B8 | Invitrogen |
| APC | B220 | RA3-6B2 | Biolegend |
| APC | CD11b | M1/70 | Biolegend |
| AF700 | AF700(CD16/32) | 93 | Invitrogen |
| APC-Cy7 | Streptavidin | - | Biolegend |
| BV510 | CD48 | HM48-1 | Biolegend |
| BV605 | CD150 | TC15-12F12.2 | Biolegend |
| Biotin | Ter119 | TER-119 | Biolegend |
| Biotin | Gr1 | RB6-8C5 | Biolegend |
| Biotin | CD11b | M1/70 | Biolegend |
| Biotin | B220 | RA3-6B2 | Biolegend |
| Biotin | CD4 | RM4-5 | Biolegend |
| Biotin | CD8a | 53-6.7 | Biolegend |
